# Perceived Symptoms in People Living with Impaired Glucose Tolerance

**DOI:** 10.1155/2011/937038

**Published:** 2011-07-12

**Authors:** Susanne Andersson, Inger Ekman, Ulf Lindblad, Febe Friberg

**Affiliations:** ^1^Institute of Health and Care Sciences, The Sahlgrenska Academy, University of Gothenburg, P.O. Box 457, 405 30 Gothenburg, Sweden; ^2^School of Life Sciences, University of Skövde, P.O. Box 408, 54128 Skövde, Sweden; ^3^University of Gothenburg, Centre for Person-Centred Care (GPCC), P.O. Box 457, 405 30 Gothenburg, Sweden; ^4^Department of Public Health and Community Medicine/Primary Health Care, The Sahlgrenska Academy, University of Gothenburg, P.O. Box 457, 405 30 Gothenburg, Sweden; ^5^Department of Health Studies, Faculty of Social Sciences, University of Stavanger, 4036 Stavanger, Norway

## Abstract

The aim of the study was to identify symptoms in people with impaired glucose tolerance (IGT) and describe their experiences of living with the symptoms which they related to their condition. Twenty-one participants, from a cross-sectional population-based study, diagnosed as having IGT, were invited for an interview. The interviews were analyzed in two phases by means of a manifest and latent content analysis. The narratives included seven categories of symptoms (and more than 25 different symptoms) presented by the respondents. This study shows that symptoms such as the patient's own interpretation of different perceptions in the body must be considered, as well as signs and/or objective observations. Symptoms ought to be seen as complementary components in the health encounter and health conversation. The results of this study indicate that health professionals should increase their awareness of the balance between the implicit and the explicit bodily sensations that individuals communicate. Further studies are needed.

## 1. Introduction

Living with impaired glucose tolerance (IGT) means living with an increased risk of developing diabetes mellitus type 2 (T2DM) and is preceded by a long period without symptoms, which is why IGT often remains undetected for a long period of time [1, 2]. At the same time, the prevalence of T2DM is predicted to increase in future decades [3–5], thus emphasizing the importance of identifying additional aspects of understanding what it means to live with IGT. 

The diagnosis of IGT is based on blood glucose level and determined by OGTT, an oral glucose tolerance test. Impaired glucose tolerance is defined as the two-hour value at OGTT 7.8–11.0 mmol/L and fasting plasma glucose <7 mmol/L according to WHO guidelines [6]. A significant number of patients with IGT already have typical diabetes complications at the time of diagnosing T2DM [7], but knowledge about their illness experiences such as emotional distress are rarely described [8]. Suitable prevention strategies are needed, including both symptoms and signs [9]. It is therefore of significance to describe if any symptoms are perceived by persons diagnosed with IGT.

Symptoms refer to the patient's own interpretation of different sensations in the body: illness, while signs on the other hand are related to objective observations: disease [10]. Signs are abnormalities in the structure and function of body organs and systems and can often be identified by signs of bodily disorder such as oedema, high blood glucose, or large amounts of urine [10]. Symptoms are a large focal point in health conversations between patients and caregivers, patients communicating their symptoms in order to better make themselves understood [11, 12].

Today it is possible to identify at-risk persons with glucose levels above normal but below the recognised threshold for diabetes, impaired glucose tolerance (IGT), and impaired fasting glucose (IFG), collectively known as prediabetes, and thereby the future risk of developing T2DM. We also know that lifestyle modification programs can reduce the risk of developing T2DM by over 50% in individuals with impaired glucose tolerance [13]. The consequences of expanding technical and medical knowledge are a challenge to modern health care practice, creating new and complex issues [14]. According to Troughton et al. [15], patients, those at risk of and identified as prediabetes feel uncertain about the diagnosis, the physical consequences, and how to cope. This complexity indicates that, to identify and support these individuals, it is necessary to know more about how symptoms are perceived by persons in early stage of the disease when diagnosed by IGT. 

The current literature to date has not examined the way that people such as those with IGT/prediabetes express and experience their symptoms at an early stage in the disease. Koopman et al. [16] studied newly diagnosed persons with T2DM and their experience of transition from prediabetes to T2DM, focusing on how symptoms are interpreted and related to prediabetes/T2DM. The results show that individuals interpreted the symptoms and often related it to other causes. They did not know about possible current symptoms and saw obstacles to diagnosis. As Adriaanse et al. [17] state, a person recently struck by T2DM may experience it as a heavy burden, which increases the likelihood of associating that which is experienced in the body as symptoms more often than is the case with nondiabetic persons. Moreover, in a study about living with prediabetes, Andersson et al. [18] show that this means living on the borderline of either being healthy or suffering from T2DM. Being on the borderline and balancing between possibilities and obstacles was interpreted as a distressing feeling. This feeling can change to one where either possibilities or obstacles are faced. 

Moreover, there are some studies concerning prevention/screening programs related to symptoms, followed up by questionnaires. Screening-detected T2DM patients are bothered more by symptoms of hyperglycaemia and fatigue in the first year following diagnosis of T2DM than nondiabetics [8]. Individuals with worsening glucose metabolism, such as in diabetes, report higher levels of symptom distress (Symptom Checklist DSC-R 10) than those with IGT or normal glucose metabolism [19]. Giel et al. [20] evaluated the mental health outcome of a lifestyle intervention by Symptom Checklist-90-R. After 9 months, the result suggests rather beneficial changes concerning symptoms of anxiety, depression, and overall psychological distress in people attending the prevention program. In their study, Paddison et al. [21] aimed to identify factors predicting symptoms such as anxiety and depression in people attending a diabetes screening. The screening outcome, whether positive or not, was not associated with higher anxiety and depression after 12 months. 

Thus, symptoms can be described as experienced bodily changes associated with illness [22–24]. Symptoms perceived as related to a diagnosis are not uncommon [25]. It is therefore conducive to ask persons with IGT to express experiences in their own words. The aim of this study was to identify symptoms in people with IGT and describe their experiences of living with the symptoms which they related to their condition.

The following questions were addressed to the data:

- what symptoms can be identified in people with diagnosed IGT?
- how do persons diagnosed as having IGT describe and talk about their symptoms?

## 2. Methods

### 2.1. Subjects and Context

The participants were selected from the Vara-Skövde Cohort in the Skaraborg Project, a cross-sectional population-based health survey in two communities located in South-Western Sweden. The project was carried out during 2001–2005, the number of participants totalling 2816 participants. The data collection has previously been described in detail [26]. Participants randomly selected by sex and age among residents 30–74 years old were consecutively invited to the local healthcare centre for a health examination for two visits. Inclusion criteria for the present study were IGT diagnosed by fasting and a 2-hour blood glucose and oral glucose tolerance test according to WHO guidelines [6] along with being informed about this diagnosis at a health check. A letter was sent to 36 selected participants, inviting them to participate in an interview regarding perceived symptoms in people with IGT and their experiences of living with the symptoms. Diagnosis with T2DM was a criterion of exclusion. Twenty-one participants aged 40–65 agreed to participate. The participants varied in age and sex which was deemed relevant in tackling the question of research. 

The interviews were conducted in different places depending on the participants' choice, such as their place of work, at the healthcare centre, or at the institute of research. The interviews were audiorecorded and transcribed verbatim. The opening question was the following: Please tell me what happened since you first became aware of having elevated blood glucose level? This was followed up by asking: Could you expand on that? The participants were asked to describe in detail symptoms which they related to their IGT. The terms “perceive” and “perception” are/were used because they refer to the participants' ways of expressing that which is experienced in the body related to living with IGT, its description, and interpretation. During the interview, previously known symptoms related to other chronic diseases were described and revealed but not explored further. 

### 2.2. Data Analysis

The analyses were conducted in two parts to answer the two separate research questions. The text was analysed by means of content analysis, including a manifest and latent analysis [27]. The interviews were read several times to ensure immersion in the data. All interview text was considered as a unit of analysis [27]. The first part, the manifest analysis, addressed the question regarding possible symptoms that could be identified and related to IGT on a level of content, and meaning units were selected. The text was condensed, and a list of symptoms was established. Subcategories were identified and grouped into categories.

The second part of analysis, a manifest analysis [27], addressed the question of how the participants talked about their symptoms on a contextual level. All text about symptoms related to their IGT by the participants was extracted and brought into one text, a unit of analysis. The symptoms identified in the first part of the analysis were used as a frame for sorting the content related to how the participants talked about their symptoms. The text was condensed, abstracted, and labeled with a code. Categories and subcategories were identified. A latent (interpretative) analysis was subsequently conducted in order to create a deeper understanding of the meaning of the identified symptoms. This resulted in a comprehensive understanding of the whole. 

### 2.3. Trustworthiness

This study addressed people with lived experiences of perceived symptoms that they themselves related to IGT, and the amount of data was considered rich, contributing to variety in the phenomena of interest. The atmosphere during the interviews was relaxed, and the interviewer tried to encourage the participants to narrate at their own pace. To ensure trustworthiness, there was awareness of and openness to misunderstandings resulting from the interviewer's own preunderstanding [28]. According to Graneheim and Lundman [27], a text always involves some degree of interpretation and multiple meanings. For this reason, the authors maintained an ongoing dialogue during the analysis to ensure consistency in descriptions and interpretation. The interpretations were not verified verbally by the participants. Verification of the interpretations was built into the analysis related to a search for coherence among the different parts of the analysis. The results involve concrete descriptions and interpretations which hopefully enable the reader to understand how the results have been gathered. 

### 2.4. Ethical Considerations

The study was approved by the Research Ethics Committee at the Faculty of Medicine, University of Gothenburg, Sweden (diary no. Ö 111-1). Written informed consent was obtained from each participant, and they were assured of confidentiality. The interviews were conducted in privacy, and confidentiality and anonymity were ensured. The audiotaped interviews were coded and contained no personal data. According to the interviews, although participants did not experience illness which had led to contact with health services, they were in a situation involving increased risk of disease. The possible harm caused by the study must be valued against the benefits. Thoughts and concerns of an existential nature may have been raised when questions were asked about perceived symptoms. On the other hand, the interviews opened for possibilities to verbalise issues/thoughts and concerns related to living with IGT. Many participants discussed issues directly after completion of the interview, but noone requested further contact.

## 3. Results

The results are structured in correlation with the two research questions:

- what symptoms can be identified in people with diagnosed IGT?
- how do people diagnosed as having IGT describe and talk about their symptoms?

### 3.1. Symptoms in People with Diagnosed IGT

**Figure 1:**
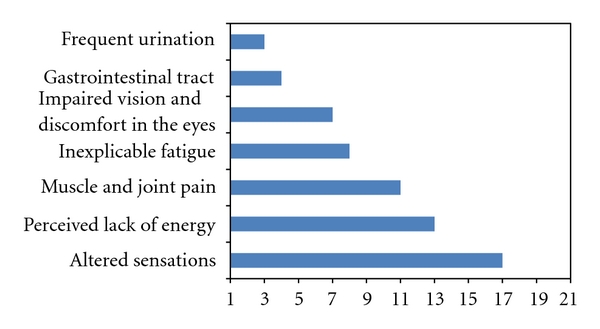
Frequencies of symptoms among individuals with IGT. Participants (*n* = 21) could report more than one symptom.

Symptoms in people with diagnosed IGT are presented in Table 2. Some of the participants had other diseases with already familiar and known symptoms, but these are not included in Table 2. The frequencies of symptoms in people having the condition are presented in Figure 1.

### 3.2. How Do People Diagnosed as Having IGT Describe and Talk about Their Symptoms?

#### 3.2.1. Altered Sensations

**Creeping Sensations and Tingling**

The participants provide a rich description of annoying, creeping sensations, and tingling. The symptoms are especially prominent in situations that are related to inactivity or fatigue, mostly in the evening.

*Yes—when I stretch and change position all the time when I go to bed. Especially if I'm lying down then. /Yes, when it's bedtime, that's when it starts (the creeping sensation (p no 1))*

These sensations result in the person doing some type of activity to relieve the discomfort, for example, stretching and bending their legs, getting up and walking around, or getting someone to help them stretch their legs properly. The ailment appears to intensify with time and become more obvious.

**Numbness**

Numbness is another common symptom. Participants say it “may” affect arms, hands, feet, and toes—uncertainty is apparent in this use of the word “may.” The symptom is described in terms of an affected part “going to sleep” or “going numb on and off.” An impoverished sense of feeling is experienced which does not go away but persists as an unpleasant, irritating sensation.

*My feet go numb, yeah … it might be numbness, poor sense of feeling in my toes and the sides—that's what can happen to me … (p no 21)*

These ailments are most apparent at night or early in the morning. The symptoms are perceived to be troublesome and lead to some kind of physical activity or movement in order to counteract or relieve them.

**Cramping and Increased Sensitivity**

Cramping sometimes occurs, the participants describing various ways of dealing with it. This might involve anything from “stretching” to rubbing in balms and/or massaging the area themselves or getting someone to help them.

Increased sensitivity is also described by the participants in terms of vague sensations and feeling cold, even though, for example, their feet are warm. Such vague signals make it difficult to pinpoint what they relate to.

*Yes, I have these creeping sensations and I get so cold … I get really cold feet but they're red hot. So you think they're ice cold but they're really warm (p no 18)*

#### 3.2.2. Perceived Lack of Energy

**Shivering and Shaking**

A very clear and apparent symptom which is directly related to the metabolism of blood glucose is shivering and shaking in between meals. Some participants describe this as a very disturbing symptom which affects their ability to work or even to drive a car. These symptoms are relieved by food intake.

*Yeah, especially if I—say I'm out cutting the grass and might be out there for an hour, and maybe I haven't eaten for a while. So partly I … Not enough food and then physical exertion—I can come over all shaky and then I have to go in and have something—milk or buns or something like that. So that this—especially this—but I've felt like this for years and years—yeah, so that this low, it goes away. That's what I feel (p no 13)*

A clear connection exists between cause and effect, affecting how an individual plans their day. Participants describe preventative measures for symptoms, such as eating lunch before driving home or taking something with them as a bag with yoghurt and fruit before going out.

**Lethargy and Cold Sweats**

Lethargy, cold sweats, and general discomfort in the form of feeling faint or dizzy are also described. Participants relate these symptoms to lack of energy, not enough food, or going too long between meals and describe them as low or falling blood glucose. Such symptoms can be perceived as very unpleasant, as one participant relates.

*I feel so dizzy almost like I find it difficult to … yes, control. Or, yeah, I can get a bit loopy, or whatever you want to call it. Not badly but I don't feel well, and then I know my blood glucose is low (p no 12)*

A number of severe symptoms such as shivering and shaking in between meals, feeling faint or dizzy are described and interpreted by the participants as being caused by low blood glucose or a lack of energy. The physical sensations that the participants attribute to low blood glucose are self-reported, and the severity of symptoms varies from that of a weak sense of feeling off form to severe symptoms which prompt immediate remedial action, such as eating something.

#### 3.2.3. Muscle and Joint Pain

**Muscular Pain and Stiffness**

Participants describe pain in a detailed and variegated fashion, at times relating it to some specific activity, such as standing still. Physical activity and inactivity may contribute. The pain itself may be of an intense or more moderate nature, located in muscles and joints, and sometimes perceived as a burning to stinging sensation.

*And it is … but it really is and pain in my … calf muscles and ligaments and everything, especially if I've been standing up. Been stood up still during the day—that's when there's no getting away from it, you know (p no 18)*

Participants describe diverse experiences of muscle and joint pain, often vocalizing an underlying assumption regarding its cause and a variety of explanations. It is notable, however, that, although participants do not consider their muscle and joint pain to be completely normal, they do not consider it troublesome enough to seek medical attention.

**Joint Pain**

Afflicted joints are variously described as “sore,” “weak,” or “feeble,” pain affecting both major and minor joints. Participants describe these ailments as possibly stemming from rheumatic or hereditary factors. Apparent, nonetheless, is that such pain is not considered to be normal or something affecting other people.

*Well, my joints ache, you know … they're weak and poorly—I can feel them and apparently you're not supposed to be able to do that (p no 16)*

Nevertheless, when participants do find their symptoms troublesome enough to seek medical attention but then receive no medical explanation, they voice their own suspicions as to the cause of their pain since there is no alternative explanation.

#### 3.2.4. Inexplicable Fatigue

**Abnormal Fatigue**

Everyone is familiar with the sense of fatigue that they may feel after exertion or a whole day at work. In this study, however, the participants do not perceive their described fatigue as ordinary tiredness. Falling asleep as soon as they sit down or just longing to go to bed is not considered completely normal.

*Yes, abnormally tired so that I, when I sit and fall asleep in front of the TV and … when you sit down quite early on in the evening and then just conk out … that's, I think it's a bit abnormal (p no 4)*

Fatigue is described as a feeling during the day that may have been experienced for a long time but is now perceived to be increasingly abnormal, sometimes affecting the desire to do anything at all, and even making everyday chores difficult.

*Yes, I get very tired in the evenings. I can't do much after work and I don't really want to do anything … just collapse. (laugh) I get angry with the plants cos they need watering and stuff … (laugh) (p no 9)*

**Increasing Fatigue**

Some persons describe their fatigue as an obstacle. Once again a link between blood glucose levels and a sense of “paralyzing fatigue” is made. There is an increased need for rest and a feeling of not being as strong as before—linked to “not having as much energy” as before.

*For a while I actually thought, I thought it was the blood glucose you got so tired from (laugh) (p no 1)*

Fatigue is perceived to be abnormal and a hindrance and impacts on daily life and the desire to do things.

#### 3.2.5. Impaired Vision and Discomfort in the Eyes

**Impaired Vision**

Some participants state that their vision has become impaired. While the idea that this is part of the natural aging process is acknowledged, there is a sense of this impairment being more pronounced than usual. Nevertheless, participants hesitate to link blood glucose levels with its effects on eye health and vision, even though relatively rapid deterioration is experienced in this regard, prompting several purchases of spectacles and visits to the optician.

*My vision is much worse, it is, and it's getting worse quite quickly because I've changed my spectacle prescription three times in just a few years (p no 15)*

Participants report that it can take time for them to adjust focus from close up to far away, their eyes requiring “recalibration” for each change. They also find the various explanations they come up with unsatisfactory and instead emphasize the importance of finding a good solution, such as a new pair of glasses. Quality of vision may even vary throughout the day. Impaired vision also affects balance and produces a feeling of instability when walking.

**Sore, Irritated, and Runny Eyes**

One participant describes his eyes as painful, another as though he has grit in his eyes.

*It's my eyes—my eyes always hurt so much (p no 20)*

Participants also report suffering from irritated, runny eyes—a variable ailment that comes and goes. Dry, irritated skin around the eyes is another abnormality described, with many different ideas being put forward as to what might cause it and a desire to find an explanation.

### 3.3. Gastrointestinal Tract

#### 3.3.1. Dry Mouth and Increased Thirst

Participants describe an increased tendency to dryness of the mouth and its mucous membranes. An unpleasant dryness of the mouth may sometimes precede thirst as the first symptom, especially during the night and upon waking, when some describe the mouth as being “extremely dry.” Dry mouth can be a troublesome ailment at other times of the day too and is sometimes associated with other related discomforts.

*I don't feel thirsty but I feel … I feel how I have pangs and soreness in my stomach, as well as this feeling of dryness in my mouth (p no 19)*

Dry mouth leads to increased thirst which participants describe as more pronounced on some occasions than others. Such thirst may be palpable and disturbing but on the whole is described as an abnormal thirst which is alleviated by drinking more water.

*I drank and drank until I became … it felt like I was absolutely bursting with water but I was still thirsty (p no 12)*

Participants may thus view increased thirst as an actual symptom based on their perception of what is normal, in comparison with other persons around them forming a frame of reference.

#### 3.3.2. Stomach and Intestines

Participants report various ill effects on the stomach and intestines due to food intake such as “loose stools,” alternately “loose/hard” or even increased gas. Other participants try to cut out food containing sugary foods or those with natural sugars and find that they feel better for it. As one participant relates.

*Yes, yes … er, I think that if I … I've noticed that if you eat sweet things then, er—before I could do that and it didn't affect me but now, if I do, I feel sick. And it disgusts me almost, real quick (p no 13)*

Knowing that sugar in various forms is bad for persons with IGT prompts these participants to react, for example, by remarking that they notice its ill effects.

#### 3.3.3. Frequent Urination

**Frequent Need to Urinate**

Participants may perceive the need to urinate several times a day as a source of irritation, regardless of whether it occurs at night or during the day. Referring to previous habits, they note there is a difference in frequency. However, frequent urination is not always taken to be a symptom of diseases; rather focus is given to how disturbing it is. As one participant comments.

*Sometimes you're constantly going (to the toilet) (p no 6)*

The results reveal a number of symptoms that cannot be dismissed, regardless of their correlation with blood glucose levels. Although participants previously regarded these symptoms as having first appeared with a rise in blood glucose level, the results of this study show that such symptoms and discomfort can be experienced before diagnosis with T2DM.

#### 3.3.4. Comprehensive Interpretation of the Meaning of Symptoms for Persons with IGT

Symptoms of varying intensity are presented by the participants, some of whom appear not to have reflected on their symptoms until the time of interview. Our interpretation of the interview data is that participants have inarticulate perceptions of and attitudes towards treating the symptoms they experience. They feel able to identify a symptom as abnormal on the basis of comparison with what they consider normal and seek explanation. Some symptoms are more distinct and therefore generally associated with a previously known diagnosis of IGT. The diagnosis may serve as confirmation and provide a possible explanation: something to relate to. When the bodily experience becomes more and more pronounced, participants begin to analyze this from a reflective stance, evaluating possible explanations in relation to preventive measures such as changed food habits or changed level of exercise. Persons decide on an explanation that is logical and relevant to them. This often involves finding a relevant strategy of preventative measures to avoid or alleviate troublesome symptoms. In summary, living the in-between stage involved perceiving changed bodily experiences, and the knowledge of having the diagnosis IGT may lead and guide ways of acting and thinking, presumably influencing ways of thinking and acting in everyday life.

## 4. Discussion

This study revealed that persons with diagnosed IGT report a range of symptoms, which is interesting considering that prediabetes including IGT is often described as a disease without symptoms [1]. The prevalence of self-reported chronic diseases among the participants in this study was low (see Table 1) which can be partly explained by the fact that the primary research question focused on symptoms that the participants related to IGT. Symptoms related to other chronic diseases were not explored further in the interviews. It is possible that other diseases have influenced and produced unexplained symptoms, which, in this case, is to be seen as a limitation of the study. However, several IGT-related symptoms described by the participants can be regarded as providing important information about living in-between being healthy and being diagnosed as having a disease. We need to understand more how to raise the awareness of T2DM in people at risk, at the same time avoiding negative effects such as labelling effects [8]. 

The fact that there is an existing diagnosis may reinforce the participants' experience and interpretation of symptoms, something which has been previously described by Kjellgren et al. [25] in patients with hypertension. According to Swedish historian, Johannisson [29], the diagnosis itself has power and highlights the difference between being healthy and sick, determining the limits to what can be considered normal and abnormal. This can be related to the distinction between disease and illness, which is discussed in medical anthropology [10]. Disease is a measurable pathological process, in contrast to illness, which is the personal experience or sensation [30]. Considering the difference between disease and illness is of importance in the care provider and patient relationship. We can assume that persons do not seek treatment for IGT. They seek help because they have symptoms which reflect how they feel, what they can do, and how they are viewed by others [30]. Persons employ different explanatory idioms grounded in everyday life despair, and care providers assume that symptoms are to be interpreted as reflections of disordered somatic (e.g., biochemical, neurophysiologic) processes [31]. The task of the care provider is therefore to decode the patients' symptoms to their biological referents in order to give an explanation [32]. However, many symptoms are poorly related to any obvious somatic disorder [33]. Medically unexplained symptoms are the reason why a large proportion of people seek health care today [34, 35]. Mishler [36] points to the fact that patients sometimes incorporate a “medical voice” when trying to explain their symptoms. Patients and care providers need to trust patient assessment to a greater extent. We know, for example, that a patient's self-reported intensity of symptoms, which may be nonconcordant to the physician's or nurse's assessment, predicts mortality up to five years [37]. The burden of symptoms varies, and the nurse's role as part of the health professional team is to comfort and if possible alleviate this burden, not give a preliminary explanation of the pathological processes. For this reason, knowing about the symptoms that persons with IGT experience is of significance when developing health educational sessions.

The results show that the experience of various symptoms is not always related to the abnormal blood glucose value. We know from several chronic conditions that pathological markers such as blood glucose and symptoms do not have a one-to-one relationship; signs can be identified without experience of symptoms, and similar degrees of organ pathology can generate quite different symptoms [37]. However, one interpretation is that the specific symptom mirrors a bodily sensation that is experienced and is nearly unaware. First, it appears as a strange or unknown sensation of an indistinct character which is not directly recognizable. It is like a feeling in the body shared by others but not talked about [38]. This may be related to Gadamer's [39] statement “health is silent” which means that, if no symptoms present themselves, health is a stable state. In this state, the presence of symptoms need not be excluded, but they are not experienced as adverse or threatening. Another way of talking about symptoms is to refer to the experience as something natural and therefore nothing to worry about [11, 40].

The participants also describe phases when symptoms become more and more evident, prompting them to seek an understanding and therefore an explanation [41]. As Hansson Scherman and Löwhagen [12] show, individuals develop their own way of understanding the illness over time, one which may not necessarily coincide with clinical practice or medical approaches to illness. We argue that unless there is dialogue between care provider and patient regarding the latter's experience of illness, there will be no improvement in treatment [12, 42]. Symptoms are something that individuals perceive and relate to sickness [43]. What is initially considered to be normal could subsequently be considered abnormal if it is related to an abnormal blood glucose value. Symptoms may confirm the knowledge of the measured increase in glucose and then be seen as an objective explanation for the symptoms. 

We would like to point out that the relationship between blood glucose level and reported symptoms is not a focus in this study. In contrast to a comparative study, the results of this one are solely descriptive. Symptoms are subjective and care providers should recognize and value their importance to patients. As Hansson Scherman and Löwhagen [12] argue, identifying the patients' conceptions of illness is of ultimate concern for evidence-based work in health education. Bodily experiences as described in this study support the argument presented by Hansson. They are expressed feelings and a cry for help, reflecting not only the physical aspects of the disease but also the associated impact on the patient's lifestyle, anxiety, depression, and expectations. For these reasons, symptoms will vary markedly amongst patients and within the same patient from one occasion to another. We argue that it is important to see these different denominations in order to understand the range of symptoms. Since everyone is unique in their way of expressing a specific symptom, it is important to render the descriptions in as tangible way as possible; consequently, the narrative in this study rests on a hermeneutic basis [44]. The illness narrative is a basis for understanding each person, at the same creating a basis for the dialogue between care provider and patient. In the health conversations, we encounter individuals who have something to tell, and as health professionals we interpreted their stories according to the context, culture, and environment. It is about understanding and explaining that which is different and has to be learned. Usually T2DM is preceded by a long period of hyperinsulinemia and is detectable several years before the condition may turn into T2DM [45]. Similarly, symptoms that are related to IGT may evolve gradually and eventually cause symptoms similar to a diagnosed T2DM. The results of this study indicate that individuals with IGT experience several symptoms that they themselves associate with blood sugar level.

### 4.1. Limitations of the Study

This study is a part of a larger study: the Skaraborg project based on a random sample of a population. These participants with IGT based on OGTT are otherwise hard to find. It is possible that other diseases have influenced and produced unexplained symptoms, but this was not explored further. The group of twenty-one subjects may be considered small, but the data contained rich and detailed information. The participants thus had experience of the research phenomenon and expressed willingness to participate in the study, constituting valid criteria. 

### 4.2. Conclusion

This study shows that symptoms, such as the patient's own interpretation of different sensations in the body must be considered, as well as signs and objective observations [10] and ought to be seen as complementary components in the health encounter and health conversation. To our knowledge, it is not feasible in primary health care settings to advocate a routine of OGTT tests [9]. The result of the study supports the opportunistic screening of plasma glucose on broad indications and vague symptoms such as fatigue in primary health care. Commonly, the primary issue in the health encounter is not the relation between plasma glucose value and degree of symptoms. We argue that the health dialogue should focus on listening to the individual's experience and trying to identify the rationale behind his or her way of understanding [42, 46], allowing it to be a complement to pathological structures and medical signs. Living with the diagnosis IGT is to live in the stage between having normal blood sugar levels and chronically elevated levels, as manifest in T2DM. The results of this study indicate that health professionals should have greater awareness of the balance between the implicit and the explicit bodily sensations that individuals communicate. Further studies are needed.

### 4.3. Relevance to Clinical Practice

This detailed description of symptoms in people with IGT can be seen as an indication for the health professional in health conversations, as well as for the individual. Of clinical relevance is increased focus on the patient's narrative and consideration of everyday language and expressions [44]. Listening to the patient in a structured way and identifying his or her thoughts and needs is caring. Knowledge about symptoms and how these are perceived are of ultimate concern when developing preventive lifestyle modification programmes. 

## Figures and Tables

**Table 1:** Characteristics of the informants (*n* = 21).

No.	Male/female *n* = 11 men, 10 women	Age at interview Mean age 53 years Range 40–65 years	Duration of IGT Mean 54 years	Other chronic diseases
1	F	44	72	None
2	F	54	40	None
3	M	40	47	None
4	F	53	47	Ulcerative colitis
5	F	62	63	None
6	M	60	56	None
7	F	59	47	None
8	M	65	42	Rheumatoid arthritis
9	F	46	43	None
10	M	42	49	Hypertension
11	M	64	53	Gastritis
12	F	46	60	None
13	F	46	45	None
14	M	46	44	None
15	M	63	81	Rheumatoid arthritis
16	F	60	78	Goiter
17	M	43	65	None
18	M	53	58	None
19	M	64	45	Coronary artery disease
20	F	51	63	High cholesterol
21	M	55	46	None

**Table 2:** Symptoms expressed by individuals with IGT.

Categories	Symptom
Altered sensations	Tingling muscles
	Numbness in legs, feet, toes
	Numbness in arms, hands, fingers
	Cramps in the legs, calves
	Burning legs, feet
	Increased sensitivity
Perceived lack of energy	Tremulousness
	Faintness
	Cold sweat
	Shaky and dizzy
Muscle and joint pain	Pain in the back, shoulders,
	arms, neck, joint,
	hips, calves
	Rigidity, stiffness
	Arthralgia
Inexplicable fatigue	Abnormally tired
	Increasing fatigue
	Need for rest
	Lack of strength
Impaired vision and discomfort in the eyes	Impaired vision
	Dry eyes
	Irritated, watery eyes
Gastrointestinal tract	Increased thirst
	Dry mouth
	Gas, constipation
	Diarrhoea
Frequent urination	Nocturnal urination
	Large amounts of urine
	Frequent urination

## References

[1] Harris MI (1993). Undiagnosed NIDDM: clinical and public health issues. *Diabetes Care*.

[2] Evans P, Langley P, Gray DP (2008). Diagnosing type 2 diabetes before patients complain of diabetic symptoms—clinical opportunistic screening in a single general practice. *Family Practice*.

[3] Amos AF, McCarty DJ, Zimmet P (1997). The rising global burden of diabetes and its complications: estimates and projections to the year 2010. *Diabetic Medicine*.

[4] Wild S, Roglic G, Green A, Sicree R, King H (2004). Global prevalence of diabetes: estimates for the year 2000 and projections for 2030. *Diabetes Care*.

[5] Shaw JE, Sicree RA, Zimmet PZ (2009). Global estimates of the prevalence of diabetes for 2010 and 2030. *Diabetes Research and Clinical Practice*.

[6] WHO (1998). *Guidelines for the Definition, Diagnosis and Classification of Diabetes Mellitus and It's Complications*.

[7] Tapp RJ, Shaw JE, Zimmet PZ (2004). Albuminuria is evident in the early stages of diabetes onset: results from the Australian Diabetes, Obesity, and Lifestyle Study (AusDiab). *The American Journal of Kidney Diseases*.

[8] Adriaanse MC, Snoek FJ (2006). The psychological impact of screening for type 2 diabetes. *Diabetes/Metabolism Research and Reviews*.

[9] Yates T, Davies M, Khunti K (2009). Preventing type 2 diabetes: can we make the evidence work?. *Postgraduate Medical Journal*.

[10] Eisenberg L (1977). Disease and illness Distinctions between professional and popular ideas of sickness. *Culture, Medicine and Psychiatry*.

[11] Salmon P (2007). Conflict, collusion or collaboration in consultations about medically unexplained symptoms: the need for a curriculum of medical explanation. *Patient Education and Counseling*.

[12] Hansson Scherman M, Löwhagen O (2004). Drug compliance and identity: reasons for non-compliance: experiences of medication from persons with asthma/allergy. *Patient Education and Counseling*.

[13] Tuomilehto J, Lindström J, Eriksson JG (2001). Prevention of type 2 diabetes mellitus by changes in lifestyle among subjects with impaired glucose tolerance. *The New England Journal of Medicine*.

[14] Adelswärd V, Sachs L (1996). The meaning of 6.8: numeracy and normality in health information talks. *Social Science and Medicine*.

[15] Troughton J, Jarvis J, Skinner C, Robertson N, Khunti K, Davies M (2008). Waiting for diabetes: perceptions of people with pre-diabetes: a qualitative study. *Patient Education and Counseling*.

[16] Koopman RJ, Mainous AG, Jeffcoat AS (2004). Moving from undiagnosed to diagnosed diabetes: the patient's perspective. *Family Medicine*.

[17] Adriaanse MC, Dekker JM, Spijkerman AM (2005). Diabetes-related symptoms and negative mood in participants of a targeted population-screening program for type 2 diabetes: the Hoorn Screening Study. *Quality of Life Research*.

[18] Andersson S, Ekman I, Lindblad U, Friberg F (2008). It’s up to me! Experiences of living with pre-diabetes and the increased risk of developing type 2 diabetes mellitus. *Primary Care Diabetes*.

[19] Adriaanse MC, Pouwer F, Dekker JM (2008). Diabetes-related symptom distress in association with glucose metabolism and comorbidity: the Hoorn Study. *Diabetes Care*.

[20] Giel KE, Enck P, Zipfel S (2009). Psychological effects of prevention: do participants of a type 2 diabetes prevention program experience increased mental distress?. *Diabetes/Metabolism Research and Reviews*.

[21] Paddison CA, Eborall HC, French DP (2011). Predictors of anxiety and depression among people attending diabetes screening: a prospective cohort study embedded in the ADDITION (Cambridge) randomized control trial. *The British Journal of Health Psychology*.

[22] Hiltunen LA, Keinänen-Kiukaanniemi SM (2004). Does hyperglycaemia cause symptoms in elderly people?. *Central European Journal of Public Health*.

[23] Gregg EW, Gu Q, Williams D (2007). Prevalence of lower extremity diseases associated with normal glucose levels, impaired fasting glucose, and diabetes among U.S. adults aged 40 or older. *Diabetes Research and Clinical Practice*.

[24] Mäntyselkä P, Miettola J, Niskanen L, Kumpusalo E (2009). Persistent pain at multiple sites—connection to glucose derangement. *Diabetes Research and Clinical Practice*.

[25] Kjellgren KI, Ahlner J, Dahlöf B, Gill H, Hedner T, Säljö R (1998). Perceived symptoms amongst hypertensive patients in routine clinical practice—a population-based study. *Journal of Internal Medicine*.

[26] Larsson CA, Gullberg B, Råstam L, Lindblad U (2009). Salivary cortisol differs with age and sex and shows inverse associations with WHR in Swedish women: a cross-sectional study. *BMC Endocrine Disorders*.

[27] Graneheim UH, Lundman B (2004). Qualitative content analysis in nursing research: concepts, procedures and measures to achieve trustworthiness. *Nurse Education Today*.

[28] Lincoln YS, Guba EG (1985). *Naturalistic Inquiry*.

[29] Johannisson K, Hallerstedt G (2006). Hur skapas en diagnos? Ett historiskt perspektiv. I. *Diagnosens Makt: Om Kunskap, Pengar och Lidande*.

[30] Eisenberg L, Kleinman A (1981). *The Relevance of Social Science for Medicine*.

[31] Risør MB (2009). Illness explanations among patients with medically unexplained symptoms: different idioms for different contexts. *Health*.

[32] Ekman I, Cleland JG, Andersson B, Swedberg K (2005). Exploring symptoms in chronic heart failure. *The European Journal of Heart Failure*.

[33] Kirmayer LJ, Groleau D, Looper KJ, Dao MD (2004). Explaining medically unexplained symptoms. *The Canadian Journal of Psychiatry*.

[34] Hartz AJ, Noyes R, Bentler SE, Damiano PC, Willard JC, Momany ET (2000). Unexplained symptoms in primary care: perspectives of doctors and patients. *General Hospital Psychiatry*.

[35] Nimnuan C, Hotopf M, Wessely S (2001). Medically unexplained symptoms: an epidemiological study in seven specialities. *Journal of Psychosomatic Research*.

[36] Mishler EG (1984). *The Discourse of Medicine: Dialectics of Medical Interviews*.

[37] Ekman I, Cleland JG, Swedberg K, Charlesworth A, Metra M, Poole-Wilson PA (2005). Symptoms in patients with heart failure are prognostic predictors: insights from COMET. *Journal of Cardiac Failure*.

[38] Groven KS, Raheim M, Engelsrud G (2010). ‘My quality of life is worse compared to my earlier life’: living with chronic problems after weight loss surgery. *International Journal of Qualitative Studies on Health and Well-being*.

[39] Gadamer H-G (1996). *The Enigma of Health: The Art of Healing in a Scientific Age*.

[40] Dowrick CF, Ring A, Humphris GM, Salmon P (2004). Normalisation of unexplained symptoms by general practitioners: a functional typology. *The British Journal of General Practice*.

[41] Friberg F, Öhlen J (2007). Searching for knowledge and understanding while living with impending death—a phenomenological case study. *International Journal of Qualitative Studies on Health and Well-being*.

[42] Friberg F, Andersson EP, Bengtsson J (2007). Pedagogical encounters between nurses and patients in a medical ward—a field study. *International Journal of Nursing Studies*.

[43] Skott C (2008). Symptoms beyond diagnosis—a case study. *The European Journal of Cancer Care*.

[44] Ricœur P (2007). *History and Truth*.

[45] Martin BC, Warram JH, Krolewski AS (1992). Role of glucose and insulin resistance in development of type 2 diabetes mellitus: results of a 25-year follow-up study. *The Lancet*.

[46] Hansson A, Friberg F, Segesten K, Gedda B, Mattsson B (2008). Two sides of the coin—general Practitioners’ experience of working in multidisciplinary teams. *Journal of Interprofessional Care*.

